# Complex cesarean section: Surgical approach to reduce the risks of intraoperative complications and postpartum hemorrhage

**DOI:** 10.1002/ijgo.16094

**Published:** 2025-01-04

**Authors:** Albaro Jose Nieto‐Calvache, Diana Ramasauskaite, Jose Miguel Palacios‐Jaraquemada, Ahmed M. Hussein, Eric Jauniaux, Akaninyene Eseme Bernard Ubom, Luisa F. Rivera‐Torres, Ines Nunes, Dietmar Schlembach, Jolly Beyeza‐Kashesya, Alison Wright, FIGO Committee on Childbirth and PPH, Jolly Beyeza, Inês Nunes, Wanda Nicholson, Ferdousi Begum, Sardar Muhammad Al Fareed Zafar, Albaro Jose Nieto Calvache, Zechariah Malel, Dietmar Schlembach, Akaninyene Eseme Bernard Ubom, Diana Ramašauskaitė, Alison Wright, Sheetal Joshi, Cherie Evans, Ahmet Metin Gülmezoglu, Monica Oguttu

**Affiliations:** ^1^ Departamento de Ginecología y obstetricia Fundación Valle del Lili Cali Colombia; ^2^ Faculty of Health Sciences Universidad ICESI Cali Colombia; ^3^ Center of Obstetrics and Gynecology Medical Faculty of Vilnius University Vilnius Lithuania; ^4^ Hospital Universitario CEMIC Buenos Aires Argentina; ^5^ Airlangga University East Java Indonesia; ^6^ Department of Obstetrics and Gynecology Cairo University Cairo Egypt; ^7^ Faculty of Population Health Sciences, EGA Institute for Women's Health University College London London UK; ^8^ Department of Obstetrics, Gynecology and Perinatology Obafemi Awolowo University Teaching Hospitals Complex Ile‐Ife Nigeria; ^9^ Centro de Investigaciones Clínicas Fundación Valle del Lili Cali Colombia; ^10^ Department of Obstetrics and Gynecology Gaia/Espinho Local Health Unit Porto Portugal; ^11^ CINTESIS—Center for Health Technology and Services Research University of Porto Porto Portugal; ^12^ Department of Medical Sciences University of Aveiro Aveiro Portugal; ^13^ Vivantes Klinikum Neukölln Berlin Germany; ^14^ Mulago Specialised Women and Neonatal Hospital Kampala Uganda; ^15^ Royal Free Hospital London UK; ^16^ FIGO London UK

**Keywords:** complex cesarean section, compressive uterine sutures, postpartum hemorrhage, protocolized treatment, surgical technique

## Abstract

The incidence of cesarean section is dramatically increasing worldwide, whereas the training opportunities for obstetrician/gynecologists to manage complex cesarean section appear to be decreasing. This may be attributed to changing working hours directives and the increasing use of laparoscopy for gynecological surgical procedures, including in gynecological oncology. Various situations can create surgical difficulties during a cesarean section; however, two of the most frequent are complications from previous cesarean (myometrial defects, with or without placental intrusion and peritoneal adhesions) and the high risk of postpartum hemorrhage (uterine overdistension, abnormal placentation, uterine fibroids). Careful surgical dissection, with safe mobilization of the bladder and exposure of the anterior and lateral surfaces of the uterus, are pivotal steps for resolving the technical difficulties inherent in performing a complex cesarean section. We propose a standardized surgical protocol for women at risk of complex cesarean, including the antenatal identification of increased surgical risk, paramedian access to the pelvis, bladder dissection and mobilization, and the selection of a bleeding control strategy, considering uterine anatomy and the arterial pedicles involved in blood loss, which should be tailored to the individual case. We propose preoperative surgical planning to include consideration of the most common situations encountered during a complex cesarean, which facilitates anticipating an appropriate response for common possible scenarios, and can be adapted for low‐, middle‐, and high‐resource settings. This protocol also highlights the importance of self‐evaluation, continuous learning, and improvement activities within surgical teams.

## Synopsis

We propose a standardized protocol for the surgical management of women at risk of intraoperative complications and postpartum hemorrhage during complex cesarean section.

## 1 INTRODUCTION

The incidence of cesarean section is dramatically increasing worldwide, particularly in countries with limited healthcare resources.^1^ Although the risks associated with cesarean section generally surpass those related to vaginal birth and, despite efforts to reduce the numbers of cesareans when vaginal birth is a safe option, there are multiple factors that drive this trend. In some regions, more than 50% of pregnant women have cesarean as their primary birth option. Although cesarean section is a safe and lifesaving procedure in many cases, it can also be associated with significant short‐ and long‐term complications. The risk of obstetric complications increases with the number of prior cesareans and complications can occur during subsequent deliveries, such as uterine rupture in a trial of labor and placenta accreta spectrum (PAS).^2^ The scarring process associated with multiple cesareans includes major dehiscence of the lower uterine segment (LUS) and pelvic peritoneal adhesions. Other conditions such as obesity, uterine fibroids, and placental location within the LUS can increase intraoperative technical difficulty,^3^ which often requires expert surgical skills to avoid or manage potential life‐threatening complications, primarily intraoperative bleeding and postpartum hemorrhage (PPH).

Changes in surgical training opportunities for obstetrician/gynecologists (ob/gyn) due to working hours directives and the use of laparoscopy for most gynecologic surgical procedures over the last two decades has limited learning of the surgical skills required for complex cesarean section. In some hospitals, gynecologic oncologists are requested to assist during laparotomy, especially in cases with multiple previous cesareans,^4^ further limiting the training opportunities of the general ob/gyn. Although surgical recommendations to enhance cesarean safety have been reported,^5^ there is currently no standardized protocol to facilitate their use by less experienced surgical teams.

This article proposes a new practical protocol for the surgical management of women at risk of complex cesarean section, including useful alternatives for addressing the most frequent surgical complications.

We aim to promote the development of a preoperative management map to facilitate the prevention of complications and the resolution of complex situations during surgery.

We propose applying a bundle model,^6^ starting with prevention, using the best strategies for uterine closure, followed by the preparation of surgical teams and recognition of cases where there may be increased technical difficulty during cesarean, through a protocolized approach to complex cesarean, and concluding with outcome analysis, complication reporting, and continuous learning.

## 2 OPTIMIZING CESAREAN SECTION RATES AND CESAREAN SCAR DEFECTS

Optimizing cesarean section rates (including reducing unnecessary cesareans) is a global public health priority, with multiple initiatives by various organizations to promote vaginal births, when appropriate. FIGO has reported strategies to address this and provided recommendations.^7^, ^8^

Regarding the prevention of intraoperative complications, FIGO has published good practice recommendations on surgical techniques to improve safety during cesarean section.^9^ It is important to highlight the relationship between inadequate healing of the LUS after a previous cesarean and the occurrence of subsequent complications, such as uterine dehiscence or rupture and PAS.^10^ Within this context, different factors have been evaluated, including the type of suture (material used, locked or unlocked, continuous or interrupted, number of layers, inclusion of the endometrium, uterine zone incised).

A randomized controlled clinical trial showed that performing the first layer of hysterotomy unlocked was associated with a lower incidence of cesarean scar defects (CSDs), in particular the development of isthmocele or niche,^11^ which represents an area of permanent loss of the normal structure of the LUS.

An observational study suggested a lower incidence of CSDs when decidua incorporation was avoided during suturing of the hysterotomy.^12^ A recent randomized single blind trial found that LUS cesarean hysterotomy performed 2 cm below the vesicouterine plica in women in advanced labor was associated with a higher incidence of large scar defects detected by transvaginal ultrasound examination 6–9 months after delivery.^13^

## 3 IDENTIFYING PATIENTS AT RISK OF COMPLEX CESAREAN SECTION

Cesarean section is the most frequently performed surgery globally, with findings indicating that an ob/gyn has mastered the procedure in its simplest form and clinical outcomes improve after they have performed at least 12 procedures.^14^

This may be true for simple elective first‐time cesareans, but the situation is very different in the context of complex cesareans; for example, in patients presenting with anterior placenta previa or an LUS segment fibroid.^3^ These patients are at risk of urinary or gastrointestinal tract complications and PPH and will benefit from delivery by a multidisciplinary team with expertise in complex pelvic surgery.^3^, ^4^

Minimizing surgical risk is a key factor in analyzing maternal deaths related to complex surgical situations. Ob/gyns must identify women likely to require multidisciplinary team management in the antenatal period and, even if the individual operator has excellent surgical skills, it is still recommended that these cases are referred to institutions with additional resources, such transfusion services and intensive care, for both mother and baby.^3^ The availability of an appropriate surgical assistant, blood components, and personnel trained in urinary and gastrointestinal repair are the minimum requirements for performing a complex cesarean section.^3^ In resource‐limited settings, this means having a general surgeon and a urologist available on call to help manage complications, if necessary. Although risk categorization and the hospital network may differ by country (or by clinical setting), the clinical situation, available resources, and surgical team's skills should be evaluated. In some cases, the best option may be to defer surgery and refer the patient to another hospital if the necessary resources are not available.^15^

The most widely available tool for the preoperative evaluation of pregnant women is ultrasound imaging. The first filter in identifying those at higher risk of surgical difficulty during a cesarean is the assessment of clearly defined risk factors for complications, such as multiple previous cesareans, other previous intra‐abdominal procedures, and the diagnosis of placenta previa. All women undergoing a cesarean—especially those with these risk factors—should be evaluated first with transabdominal ultrasound, with special emphasis on the characteristics of the LUS, and then with transvaginal ultrasound if the placenta is low‐lying or previa. The signs associated with PAS have been extensively described. More recently, the sonographic signs of other high‐risk groups for complex cesarean in the absence of PAS have also been presented, with a clear recommendation to refer women predicted to be a complex case to surgical teams with specific expertise.^3^

## 4 DEVELOPING A STANDARDIZED PROTOCOL AND TRAINING FOR COMPLEX CESAREAN SECTION

Training ob/gyns to perform complex cesarean sections has become increasingly difficult since exposure to complex abdominal and pelvic surgery is limited, due in part to the increase in use of laparoscopic surgery for the majority of gynecologic conditions, including gynecologic cancer.^3^ The solutions to this are not simple and are beyond the scope of this article; however, innovative alternatives such as simulation and virtual training should be explored.

Many situations can increase technical difficulty in a complex cesarean section. Conditions such as anticoagulation and cardiovascular or pulmonary diseases increase the overall intraoperative risks, and often require management by a specialist anesthetist. Emergency cesarean performed at full cervical dilatation with engagement of the fetal head, fetal malformations such as hydrocephaly, or dystocic positions such as transverse presentation can change a simple cesarean into a complex surgical challenge.

It is impossible to describe every complex scenario; therefore, this article proposes a standardized protocol for surgical management of women with extended pelvic peritoneal adhesions, major LUS dehiscence with or without placental intrusion,^3^, ^16^ and a high risk of intraoperative bleeding and/or PPH.

Considering the surgical challenges in these cases, we propose the following steps:

1. Access to the pelvis in cases of extended peritoneal adhesions between the uterus and other pelvic organs and/or vessels.
2. Dissection and mobilization of the bladder to expose the LUS surface, which is necessary for managing large dehiscence and intraoperative bleeding.
3. Selection/application of a PPH control strategy.

### 4.1 Entering the abdomen and pelvis safely

The most frequent complication of abdominal surgery is peritoneal adhesion formation, which imposes surgical difficulty when thick or extended.^17^ In particular, bladder adhesions to the abdominal wall or a dehiscent LUS pose a risk of urinary tract injury during pelvic access. In this situation, operators will generally seek upper abdominal access, but sometimes the adhesions are so dense and extensive that this strategy is insufficient, even for expert surgeons.

**FIGURE 1:**
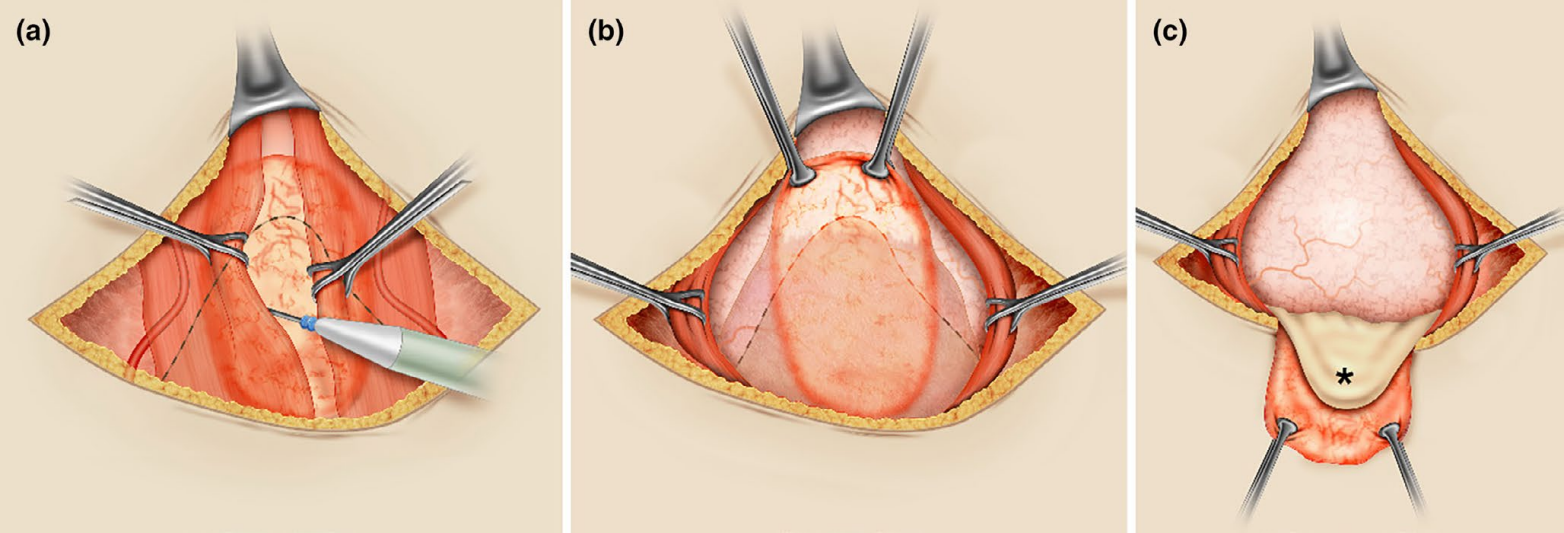
Paramedian (preperitoneal) access to the pelvis. (a) In most cases, adhesions between the abdominal wall and intra‐abdominal structures occur along the midline (orange oval area), with the bladder being the most frequently adhered organ to the abdominal wall (interrupted black line). Using a transverse suprapubic skin incision, and after dissecting the abdominal fascia from the anterior surface of the rectus muscles, preperitoneal dissection is facilitated by retracting those muscles with Babcock forceps. The dissection begins at the medial edge of each muscle, moving laterally between the peritoneum and the muscle until reaching an area free of fibrosis. Attention should be paid to the inferior epigastric vessels on the lower lateral side of the rectus abdominis muscles when the dissection is extensive. (b) Upon reaching the lateral limit of the peritoneal adhesion area to the abdominal wall, the fibrotic area can be cut and mobilized (edges retracted with Allis forceps), also mobilizing the adhered bladder (interrupted black line) without risking injury. (c) After this type of dissection, the bladder (*) can be mobilized away from the anterior surface of the uterus, facilitating the hysterotomy.

A more reproducible alternative is paramedian abdominal access^18^ using the preperitoneal plane, which is almost always accessible and facilitates finding a fibrosis‐free area behind the rectus abdominis muscles, subsequently releasing the bladder from the abdominal wall bilaterally (Figure 1). Use of this approach through a transverse suprapubic skin incision requires the surgeon to familiarize themself with abdominal wall anatomy (Video 1) and avoid injuring the inferior epigastric vessels during extensive dissection (Figure 1). Controlling small caliber blood vessels associated with the scarring process of previous surgeries is more frequent.

### 4.2 Dissection and mobilization of the bladder

One of the greatest concerns during obstetric surgery is injury to the urinary tract. Bladder injury and, less frequently, ureteric injury during cesarean or PPH control procedures are among the most frequent complications of complex cesarean section.

Accessing the avascular subperitoneal pelvic spaces is routine for specialist gynecologic oncologists,^19^ but rarely practiced by general ob/gyns. Most obstetric procedures only require developing the anterior spaces to separate the bladder from the anterior uterine surface and laterally and inferiorly mobilize the ureters.^20^

In PAS surgery, surgical staging has been described and this involves exploring the parametrial, medial paravesical, and retrovesical spaces to achieve complete bladder mobilization.^20^, ^21^ The same procedures can be performed during a complex cesarean. Bladder adhesion to the anterior uterine surface almost always occurs in the midline; therefore, lateral‐to‐medial dissection—starting in the medial paravesical space and moving toward the retrovesical space—is a safe and reproducible method to prepare for any uterine procedure (Video 2).

**FIGURE 2:**
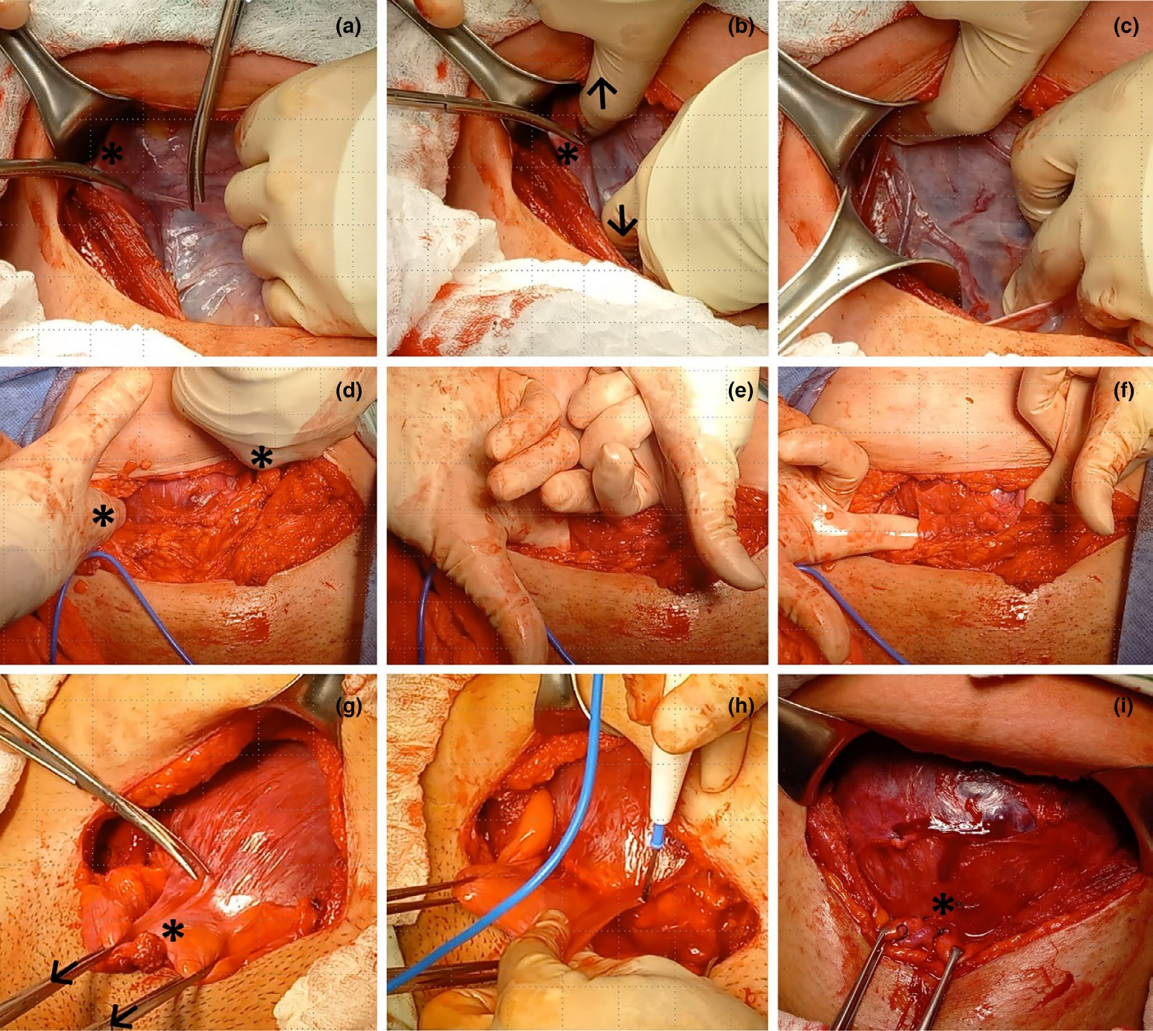
Bladder mobilization during complex cesarean section. Exposure of the anterior surface of the uterine segment is fundamental for managing the surgical challenges in a complex cesarean section. (a–c) Opening of the parametrial space. Traction of the right round ligament (*) and incision of the anterior leaf of the broad ligament (a); digital opening (arrows) of the right parametrial space in a caudocephalic direction (b); mobilization of the round ligament with a retractor and exposure of the right lateral surface of the uterus (c). (d–f) Retrovesical bypass (Pelosi maneuver). The surgeon inserts their fingers (*) into both parametrial spaces (d) and moves them caudally to reach the medial paravesical spaces (e), then directs the fingertips toward the midline, at the level of the cervix, in the retrovesical space (f). (g‐i) Dissection of the retrovesical space. Facilitated by anterocaudal traction (45 degrees from the horizontal: Arrows) of the bladder (*) with Allis clamps (g), the peritoneum is incised at the vesicouterine fold. The lateral to medial approach, starting from the previously developed medial paravesical space (h), can be performed with scissors or energy, reducing the possibility of bladder injury or uterine laceration. Complete mobilization of the bladder (i) exposes the anterior surface of the uterine segment (*) and facilitates the selection and application of interventions such as low compression sutures (Ho Cho, B‐Lynch 2), uterine artery ligation, en‐bloc resection of lesions (leiomyoma, uterine dehiscence, placenta accreta spectrum) in that topography, or even hysterectomy.

There are three possible procedures to facilitate bladder mobilization during a complex cesarean section: (1) opening of the parametrial space by applying traction to the round ligament and cutting the anterior leaf of the broad ligament to then digitally open the space; (2) retrovesical bypass (or Pelosi maneuver) by introducing the surgeon's index fingers from the parametrial space to the medial paravesical space and then to the retrovesical space; and (3) dissection of the retrovesical space facilitated with an anterocaudal traction at 45 degrees to the horizontal, using Allis clamps (Figure 2).

**FIGURE 3:**
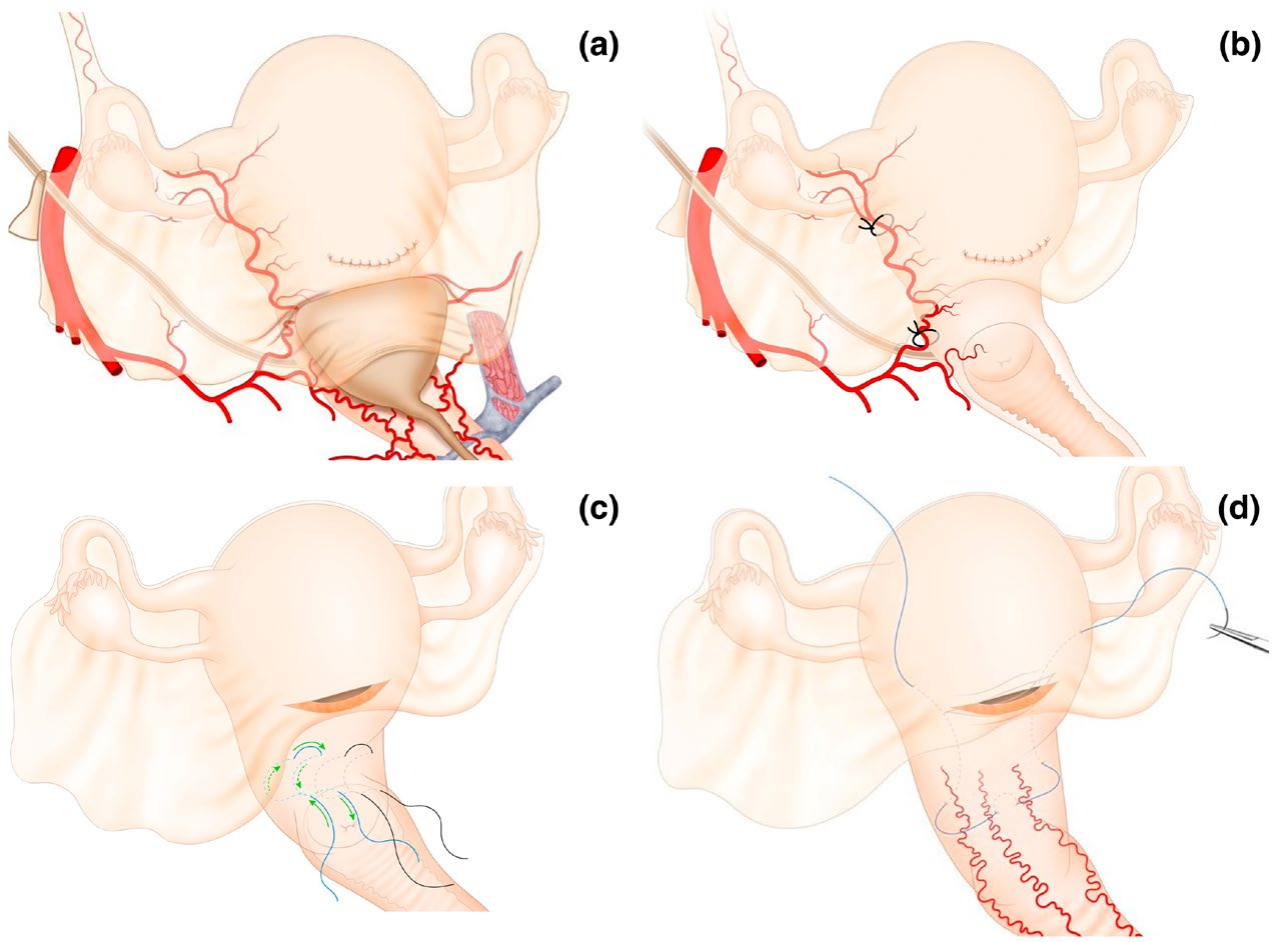
Relationship between the mobilized and nonmobilized bladder with the performance of lower uterine compressive sutures and uterine artery ligation. The position of the bladder over the anterior surface of the lower uterine segment (a) makes it clear that controlling uterine bleeding from this area requires bladder mobilization. Ligation of the uterine artery on the lateral surface of the uterine segment requires that, in addition to the uterine vessels (artery and veins), a portion of the myometrium is included in the stitch to provide support for the suture (b). A deep stitch without bladder mobilization, even when applied from the posterior surface of the uterus, can lead to bladder tear, especially in patients with previous cesarean sections and adhesions between the uterus and bladder. Compression sutures for the lower uterine segment (c, d) are indicated in abnormal bleeding secondary to placenta previa. The lower part of the uterus receives its blood supply mainly from the colpouterine vessels that ascend from the vagina, and the most well‐known lower uterine compressive sutures are the Ho‐Cho suture (d) and the B‐Lynch 2 suture (e), whose application is impossible without prior bladder mobilization.

Historically, exposure of the LUS has rarely been described as a requirement for performing uterine hemostatic procedures^22^ or during the management of patients with myometrial defects. However, if we consider the relationship between the bladder and the LUS (where bleeding associated with placenta previa occurs), the risk of bladder injury during the execution of hemostatic sutures is evident (Figure 3, Video 2).

**FIGURE 4:**
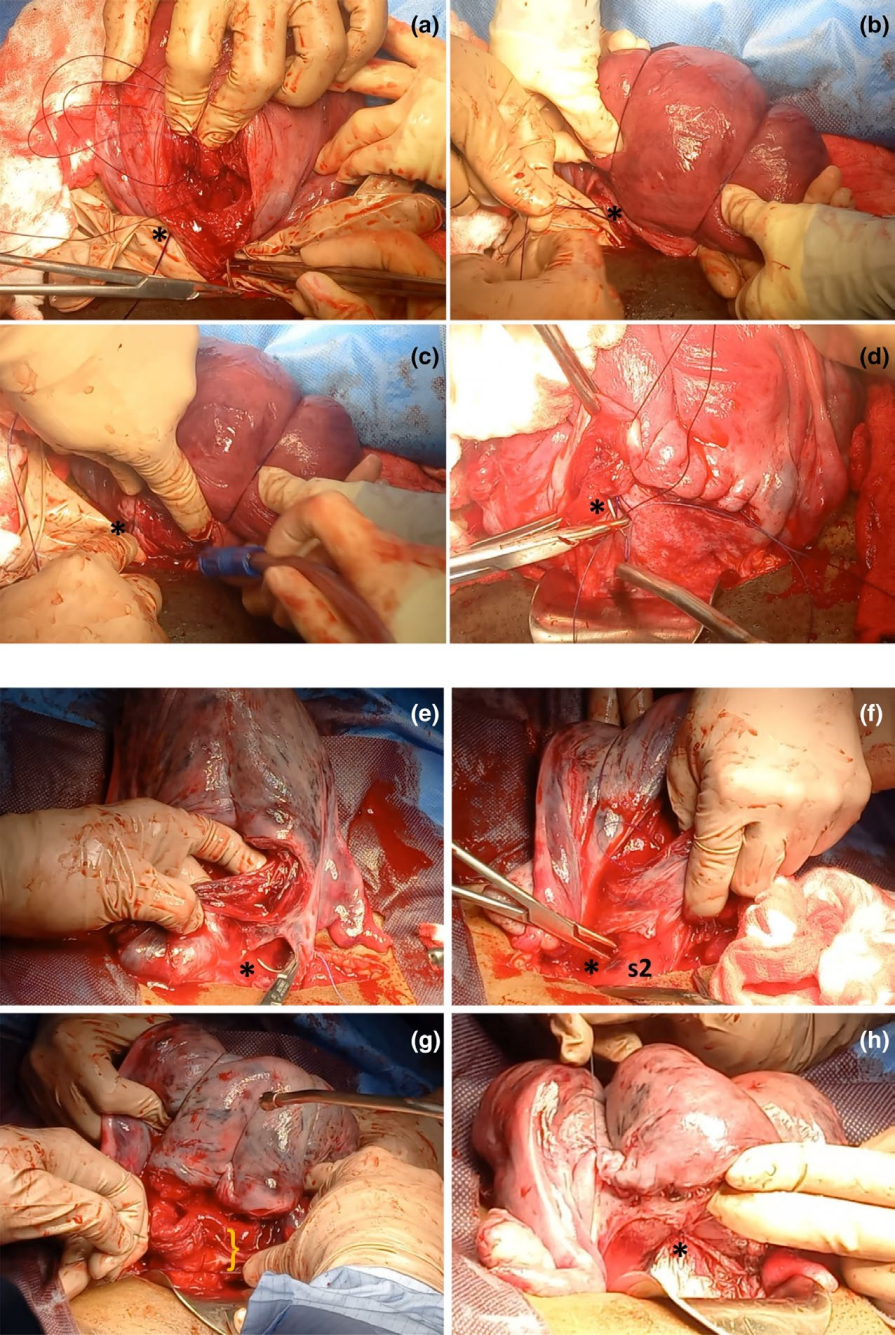
Importance of mobilization of the bladder to perform B‐Lynch suture. (a–d) Failed B‐Lynch in a patient with persistent bleeding after compression suturing and who was finally treated with hysterectomy. Application of the B‐Lynch suture without prior mobilization of the bladder implies that the initial (*) and final points of the suture are located very close to the lower edge of the hysterotomy (a) and that when compressing the uterus, the tension of the suture is supported by a very small portion of tissue (*) (b). This may cause the obstetrician to limit the force applied to the knot (*) due to the risk of lacerating the lower edge of the hysterotomy, resulting in a potentially suboptimal compressive effect (c). In these cases, the hysterorrhaphy may even pass just above the knot (*) of the compression suture (d). (e–h) Successful B‐Lynch. After dissection of the retrovesical space and exposure of the entire lower uterine segment, the first stitch of the compression suture (*) can be placed several centimeters caudal to the lower border of the hysterotomy (e). The same applies to the last stitch of the compression suture (*), which includes a significant amount of tissue in the lower part of the uterine segment in an area corresponding to sector 2 of uterine vascularization (s2), usually located behind the bladder (f). Including several centimeters of tissue at the lower border of the hysterotomy (}) allows the surgeon to exert greater compressive force with the suture without fear of tearing the tissue (g). Unlike in photo (d), in this case the knot of the compression suture (*) is located far from the hysterorrhaphy (h).

Uterine compression procedures aimed at the uterine body and fundus, such as the B‐Lynch suture, can fail if applied too close to the lower edge of the hysterotomy due to a lack of bladder mobilization (Figure 4). If the strategy chosen by the surgical team for a cesarean complication is hysterectomy, bladder mobilization is the procedure that defines the difficulty and duration of the surgery, as well as the risk of additional complications (e.g. ureteric injuries). Bladder puncture or tear can also occur during uterine artery ligation if the bladder is not initially dissected and mobilized (Figures 3 and 4, Videos 2 and 3).

### 4.3 Postpartum hemorrhage control strategy

Potentially, the most serious complication of complex cesarean section is PPH during or after the surgical procedure. Although other significant complications can occur (urinary, gastrointestinal, infectious, and many others), uterine bleeding can be so profuse to be fatal, if appropriate measures are not applied to prevent and to treat major blood loss.

Recently, a consensus statement was published, involving a panel of physicians with expertise in obstetrics, anesthetics, hematology, and transfusion medicine, convened by the Network for the Advancement of Patient Blood Management, Hemostasis and Thrombosis (NATA) in collaboration with FIGO, the European Board and College of Obstetrics and Gynecology (EBCOG), and the European Society of Anaesthesiology and Intensive Care (ESAiC) regarding patient blood management (PBM) in obstetrics.^23^

PBM is the timely application of evidence‐based medical and surgical concepts, designed to maintain hemoglobin concentration, optimize hemostasis, and minimize blood loss to improve outcomes. PBM implementation in obstetrics involves the timely application of strategies aimed at optimizing perioperative erythropoiesis (Pillar 1); minimizing surgical and nonsurgical (iatrogenic) blood loss and correcting coagulopathy (Pillar 2); and supporting the woman while appropriate treatment is initiated, including the application of restrictive transfusion thresholds (Pillar 3).^24^, ^25^, ^26^ In the following section, Pillar 2 of PBM during complex cesarean is overviewed, regarding the optimal surgical strategy and techniques to control bleeding.

In any part of the body, the key to managing bleeding is understanding the blood supply of the corresponding organ/tissue.^27^ Traditional knowledge of uterine blood supply among ob/gyns includes the uterine artery and ovarian artery as the primary arterial pedicles.^28^

**FIGURE 5:**
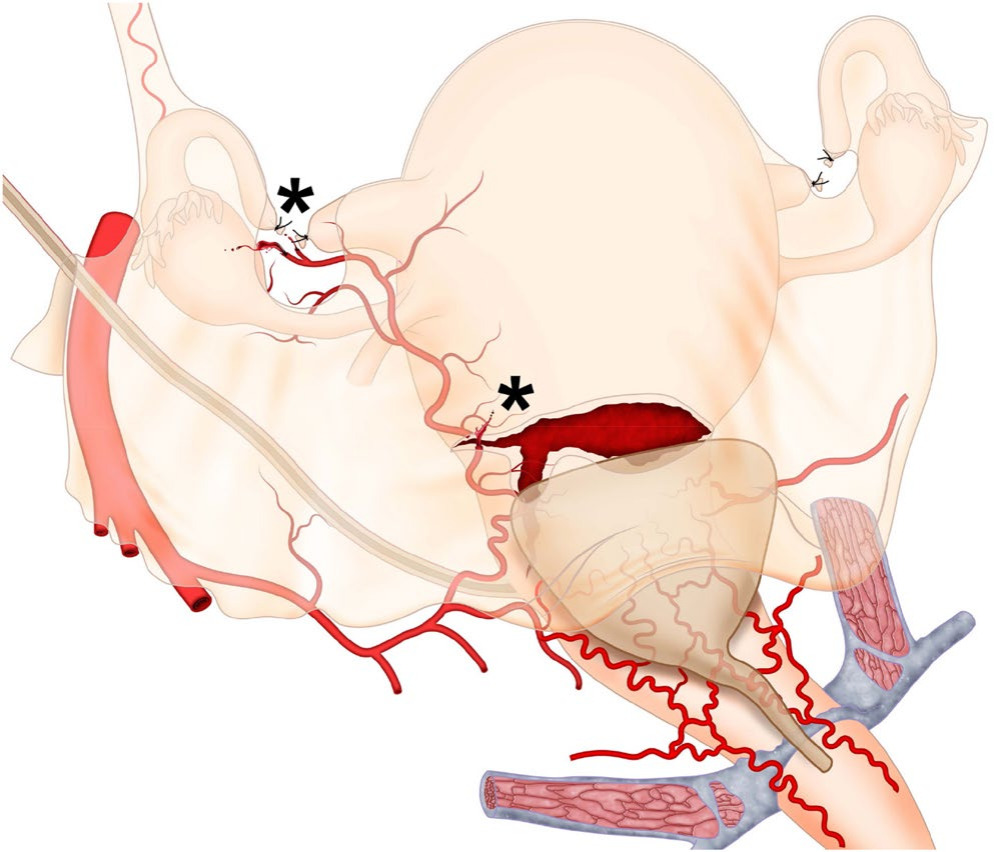
Broad ligament hematomas and hysterotomy tears. The most common bleeding sites in broad ligament hematomas are the lateral ends of the hysterotomy (due to persistent arterial bleeding from branches of the uterine artery) and the area where surgical sterilization is performed (due to bleeding from branches of the ovarian or uterine artery). During the exploration of hematomas, regardless of the size of the blood collection, the surgeon must specifically examine the suspected areas of bleeding origin (*) and for that it is essential to mobilize the bladder.

This description is incomplete and leads to misunderstandings as it overlooks the involvement of a third arterial pedicle that reaches the lower part of the uterus and derives from the vaginal arteries via the colpouterine vessels^29^ (Figure 5). The colpouterine arteries are not described in many traditional anatomy textbooks, most likely due to translation mistakes.^30^ However, these vessels have been described since 1833^31^ and have recently been rediscovered through studies on fresh cadavers, arteriography, and physiological research.^29^ Colpouterine arterial pedicles can be identified easily during cesarean section, clearly distinguishing between their location and the course of the uterine artery (Video 4).

Involvement of the colpouterine arterial pedicle in obstetric bleeding from the uterine body is minimal. Hence, its importance in cases of uterine atony (where the main vessel involved is the uterine artery) is likely to be minimal.

A very different scenario occurs when uterine bleeding originates from the lower segment of the uterus (sector 2 of uterine vascularization), as seen in women with placenta previa and PAS.^32^

Understanding the existence of these vessels and their importance in bleeding from sector 2 is extremely important for the surgeon because it facilitates the choice of application of the bleeding control strategy, considering the source and etiology of the bleeding.

For example, a woman with bleeding due to uterine atony that bleeds from the uterine body or fundus (sector 1) benefits from uterine artery ligature or embolization or by a simple compression suture that involves this area of the myometrium, such as the B‐Lynch 1, Hayman suture, or others that compress the uterine body. These sutures would not be useful when a woman is bleeding from the LUS due to placenta previa and/or major dehiscence, where a suture compressing the bleeding tissue—such as the B‐Lynch 2 or Ho‐Cho suture—would be more effective^33^ (Video 5). Similarly, uterine artery ligation would not be useful for controlling bleeding from the LUS and should be reserved for bleeding from the uterine body.^32^

**FIGURE 6:**
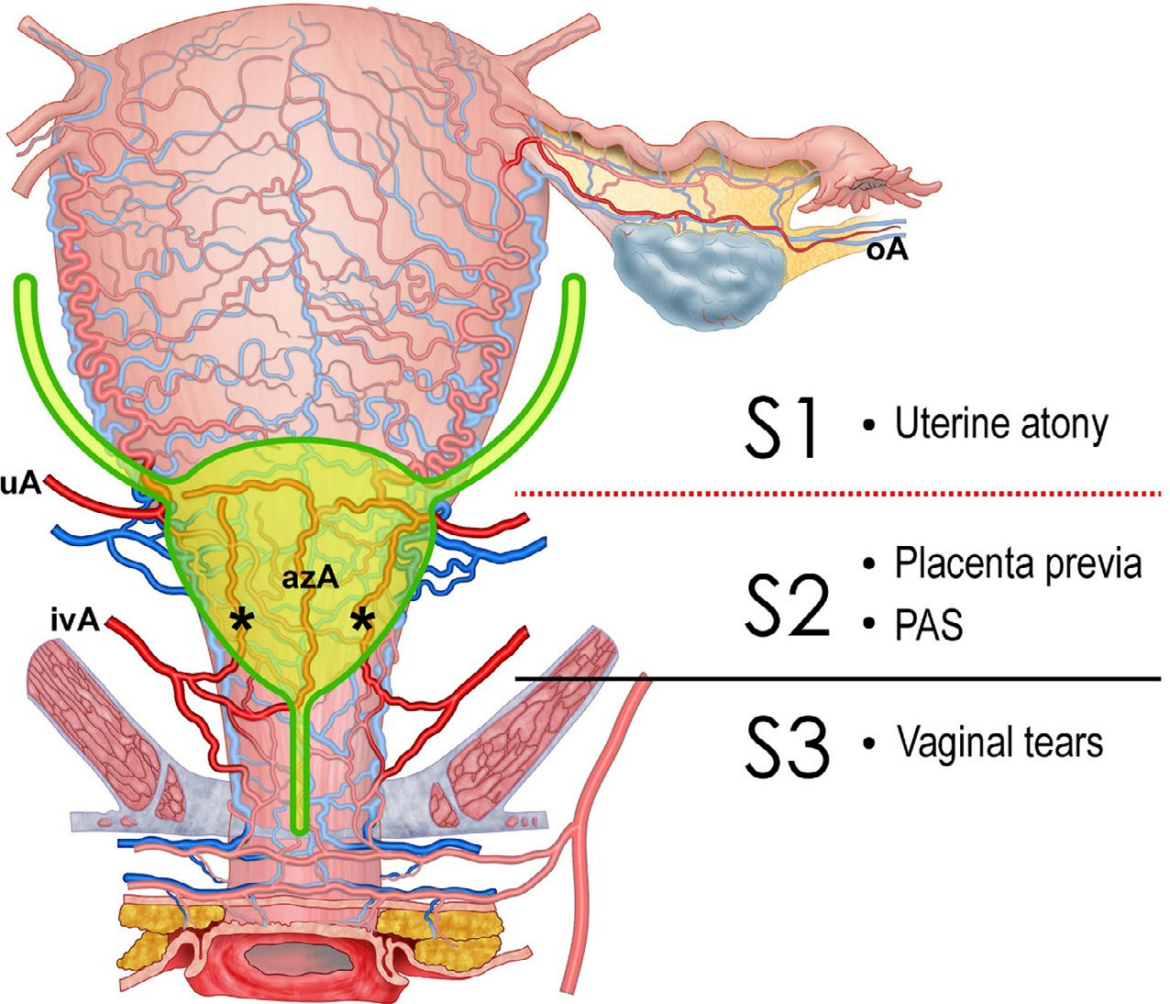
Uterine vascular pedicles. The uterus receives blood supply from three arterial pedicles. The most widely known are the ovarian (oA) and uterine arteries (uA), which supply the uterine body. The lower part of the uterus and the cervix receive arterial branches from the vagina, known as colpouterine arteries (cuA), typically identified as three main branches located in the midline (azygos vaginal artery–azA) and on either side of the anterior vaginal wall (*), reaching and anastomosing with branches of the uterine arteries. This last arterial pedicle (colpouterine arteries, branches off the inferior vaginal arteries–ivA) has been omitted in some anatomical descriptions, making it unknown to many obstetricians. Understanding these arterial pedicles allows for the establishment of two uterine vascular sectors. Sector S1 corresponding to the uterine body is primarily supplied by the uterine artery (uA) with a minor contribution from the ovarian artery (oA). Easy access to the vessels supplying this sector facilitates the control of bleeding from this part of the uterus. Conditions such as uterine atony can be successfully treated with multiple therapeutic options (uterine artery ligation, compression sutures, uterine artery embolization, etc.). Sector S2 corresponds to the lower uterine segment and cervix, receiving its main blood supply from the colpouterine arteries. Its subperitoneal and retrovesical location creates greater difficulty in controlling bleeding from this area (e.g. secondary to placenta previa or placenta accreta spectrum–PAS), necessitating bladder mobilization to perform procedures such as low compression sutures (B‐Lynch 2 or Ho‐Cho). The reference point separating sectors S1 and S2 is the peritoneal reflection (red dotted line), which is clearly visible during cesarean section when the bladder is empty. During prenatal ultrasonographic examination (or in magnetic resonance imaging) with a full bladder, the reference point is the midpoint of the posterior wall of the full bladder. In the female genital tract, a third vascular zone (sector S3), corresponds to the vagina, supplied by vaginal arteries, branches off the internal pudendal artery (originating from the posterior division of the internal iliac artery). Controlling bleeding from this zone (e.g. in complex vaginal tears associated with compound fetal presentations or instrumental vaginal delivery) demands exposure of the external surface of the vagina by dissecting the prevesical space (Retzius).

Other situations that benefit from anatomical knowledge of the three uterine arterial pedicles are uterine tears (associated with hysterotomy, uterine rupture, or other causes) and postoperative hematomas. Controlling bleeding in these situations can be facilitated when the surgeon clearly understands which arterial pedicle supplies the affected area (Figure 6), since most cases occur in the lower anterior wall of the uterus.^34^

A good example is a broad ligament hematoma, which often presents as a maternal drop in blood pressure and in hemoglobin level in the immediate postoperative period, with a transient response to volume replacement and a return of hypovolemic symptoms due to persistent arterial bleeding (Video 3). Although these hematomas can be large and distort the pelvic, subperitoneal, or retroperitoneal anatomy, the surgeon must remember that despite the extensive spread of the hematoma (sometimes reaching high portions of the posterior abdominal wall), treatment should focus on the source of the bleeding, which should only be the area operated on during cesarean section (Figure 6). Most of these hematomas originate at the lateral ends of the hysterotomy,^35^ where branches of the uterine artery are injured.

Another possible bleeding point is the area operated on during surgical sterilization, primarily the mesosalpinx, with involvement of terminal branches of the uterine or ovarian arteries. In both cases, the surgeon should explore the hematoma, drain accumulated blood, and specifically review the area manipulated during the initial surgery, until an active bleeding source is found. Even if judged to be low volume at the time, this should be controlled with a suture of the bleeding vessel before closing the abdomen.

Knowledge of the three uterine arterial pedicles and the two vascular sectors differentiated by their relation to the peritoneal reflection (Sector S1 above the reflection and Sector S2 below) facilitates the surgeon's identification of the bleeding focus and therefore the choice of the most appropriate treatment strategy (Table 1 and Figure 6).

**TABLE 1:** Hemostatic methods according to uterine irrigation areas.

Vascular uterine sector*	Arterial pedicles involved	Compressive sutures	Vascular ligature useful	Useful endovascular procedures	Endocavity compressive procedures	Other external compression procedures
S1	UA, OA	Body and fundal uterine compressive sutures**	UA	UA	Intrauterine balloon tamponade	External elastic wrapping
S2	UA, VA, VaA	Lower uterine segment compressive sutures***	CUA	Highly selective through pudendal internal artery branches	Vaginal balloon tamponade	No

*Note*: S1 corresponds to the body, fundus, and upper part of the uterine segment. S2 corresponds to the lower uterine segment. During surgery, access to the S2 region is only possible after dissecting the retrovesical space. The most frequent condition associated with S1 bleeding is uterine hypotonia. Placenta previa is the main cause of S2 bleeding. *S3 vascular region corresponds to the middle and upper thirds of the vagina (Figure 6) and can be the source of extrauterine postpartum bleeding, for example, after precipitous labor, compound presentations, or non‐recommended obstetric maneuvers such as fundal pressure (Kristeller meneuver). Vaginal arteries may be lacerated, making control via vaginal access challenging. We did not include description of this sector in the table to focus the reader's attention on the most common sources of bleeding related to complex cesarean delivery (S1 and S2 sectors). **The most recognized suture for S1 is the B‐Lynch suture, but many others exist that are primarily designed to compress the upper part of the uterus (e.g., Hayman, Pereira, and others). ***The most recognized sutures for S2 are the B‐Lynch 2 (transverse B‐Lynch) and the Ho‐Cho suture.

Abbreviations: CUA, colpouterine artery; OA, ovarian artery; UA, uterine artery; VA, vesical artery; VaA, vaginal artery.

Regardless of the etiology of the bleeding, technical difficulty, or available resources, the immediate priority is to stop the hemorrhage promptly (primary hemostasis) to prevent metabolic deterioration and while applying the definitive control strategy (secondary hemostasis).

Most relevant management guidelines have described a sequential approach with increasingly complicated interventions as time progresses or blood loss volume increases.^22^

**FIGURE 7:**
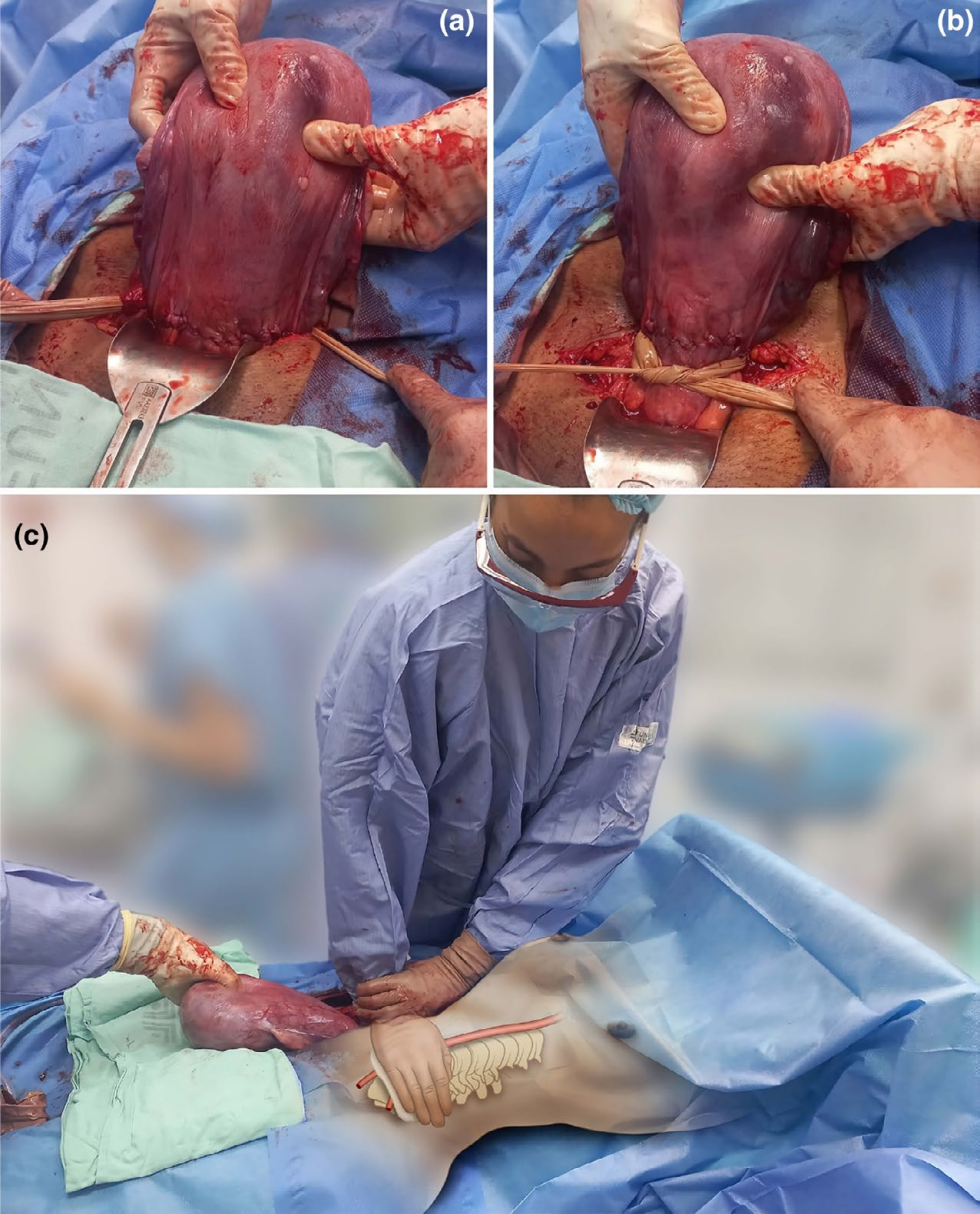
Immediate temporary control of bleeding during cesarean section. Regardless of the cause of uterine or pelvic bleeding, there are options for immediate and temporary hemorrhage control (primary hemostasis). In cases of uterine bleeding, the tourniquet with a sterile latex glove (a) applied below the level of bleeding (b) allows for bleeding cessation while other definitive control strategies are applied (secondary hemostasis). Manual compression of the infrarenal aorta (zone 3 of the aorta) provides immediate control of pelvic bleeding of any etiology (primary hemostasis). A healthcare professional positions themselves beside the patient and, after locating the aorta by palpation, compresses it against the lumbar vertebrae with the weight of their torso, positioning their arm at a 90° angle to the horizontal (c), with the elbow fully extended. A step stool should be used to achieve this position and maintain it as long as necessary (up to 60 continuous minutes without significant metabolic impact) so that surgeons can apply other definitive bleeding control interventions (secondary hemostasis).

A potentially preferable alternative is immediate control of blood loss, with simple and universally available measures such as internal^36^ or external^37^ aortic compression or uterine tourniquet (Figure 7). This way, the surgical team can arrange for available resources without the pressure of each passing minute worsening the patient's condition.

## 5 ANALYZING RESULTS AND LEARNING FROM EXPERIENCE

There is no easy or quick way for a surgical team to develop the skills necessary to handle the challenge of a complex cesarean section. Developing surgical competencies is a lengthy process and depends on multiple factors.^14^ Although the use of management protocols such as those proposed in this article can be helpful, there will always be cases that fall outside the parameters outlined and can only be resolved through the surgical skills of the team. There will also be cases so complex that they exceed the capabilities of the regular surgical team, and special situations such as resource shortages, overburdened services, or patient‐specific conditions that may lead to adverse events, near misses, or maternal deaths.

Analyzing the successes and failures of surgical teams is an essential strategy to improve quality of care.^38^ For surgical procedure analysis, reviewing intraoperative videos is indispensable. Post‐event learning should be routine for multidisciplinary teams responsible for managing patients suspected of requiring complex cesarean section. Debriefing formats, measurement of indicators, and other behavioral and human‐factor interventions are recommended. We advocate for a safety culture, where participants in the care process are vigilant about adherence to agreed group processes, and maintain an attitude of mutual respect, collegiality, self‐assessment, and continuous improvement.^39^

## 6 CONCLUSION

This article provides a detailed and technical overview of strategies and considerations for managing complex cesareans and for preventing PPH.

We propose antenatal risk assessment and an operative protocol, including paramedian access to the pelvis in cases of extended abdominal and pelvic adhesions; bladder dissection and mobilization in cases of major uterine dehiscence or high risk of PPH; and selection of the bleeding control intervention based on anatomical knowledge and bleeding focus topography.

Although some conditions leading to surgical complexity during cesarean section fall outside the scope of this protocol, we expect that the principles described in this article will facilitate safe and appropriate management for the majority of women undergoing complex cesareans.

## AUTHOR CONTRIBUTIONS

*Study planning*: A.J.N.C, J.M.P‐J. *Manuscript editing*: A.J.N.C., D.R., J.M.P‐J, A.H.H., E.J., A.E.B.U., L.F.R‐T, I.N., D.S., J.B.K., A.W. All authors read and approved the final manuscript.

## CONFLICT OF INTEREST STATEMENT

The authors declare no conflicts of interest.

## Supporting information


**Video 1.** Paramedian access to the pelvis.

**Video 2.** Bladder mobilization during complex cesarean section.

**Video 3.** Broad ligament hematoma, clinical presentation, and treatment guided by anatomy.

**Video 4.** Selection of vascular control strategy according to the affected uterine segment (S1 or S2).

**Video 5.** Colpouterine pedicles during cesarean section.

## Data Availability

Data sharing is not applicable to this article as no new data were created or analyzed in this study.

## References

[1] Betran AP , Ye J , Moller AB , Souza JP , Zhang J . Trends and projections of caesarean section rates: global and regional estimates. BMJ Glob Health. 2021;6:e005671. 10.1136/bmjgh-2021-005671 PMC8208001 34130991

[2] Silver RM , Landon MB , Rouse DJ , et al. Maternal morbidity associated with multiple repeat cesarean deliveries. Obstet Gynecol. 2006;107:1226‐1232. 16738145 10.1097/01.AOG.0000219750.79480.84

[3] Jauniaux E , Fox KA , Einerson B , Hussein AM , Hecht JL , Silver RM . Perinatal assessment of complex cesarean delivery: beyond placenta accreta spectrum. Am J Obstet Gynecol. 2023;229:129‐139. 36868338 10.1016/j.ajog.2023.02.021

[4] Futterman ID , Conroy EM , Chudnoff S , Alagkiozidis I , Minkoff H . Complex obstetrical surgery: building a team and defining roles. Am J Obstet Gynecol MFM. 2024;6:101421. 38969176 10.1016/j.ajogmf.2024.101421

[5] Gialdini C , Chamillard M , Diaz V , et al. Evidence‐based surgical procedures to optimize caesarean outcomes: an overview of systematic reviews. EClinicalMedicine. 2024;72:102632. 38812964 10.1016/j.eclinm.2024.102632 PMC11134562

[6] Main EK , Goffman D , Scavone BM , et al. National Partnership for Maternal Safety: Consensus Bundle on Obstetric Hemorrhage. Obstet Gynecol. 2015;126:155‐162. Erratum in: Obstet Gynecol. 2015;126:1111. Erratum in: Obstet Gynecol. 2019;133:1288. 26241269 10.1097/AOG.0000000000000869

[7] Barnea ER , Inversetti A , Di Simone N , FIGO Childbirth and Postpartum Hemorrhage Committee . FIGO good practice recommendations for cesarean delivery: prep‐for‐labor triage to minimize risks and maximize favorable outcomes. Int J Gynecol Obstet. 2023;163(Suppl 2):57‐67. 10.1002/ijgo.15115 37807590

[8] Visser GHA , Ubom AE , Neji K , et al. FIGO opinion paper: drivers and solutions to the cesarean delivery epidemic with emphasis on the increasing rates in Africa and southeastern Europe. Int J Gynecol Obstet. 2023;163(Suppl 2):5‐9. 10.1002/ijgo.15111 37807592

[9] Nunes I , Nicholson W , Theron G , FIGO Childbirth and Postpartum Hemorrhage Committee . FIGO good practice recommendations on surgical techniques to improve safety and reduce complications during cesarean delivery. Int J Gynecol Obstet. 2023;163(Suppl 2):21‐33. 10.1002/ijgo.15117 37807585

[10] Palacios‐Jaraquemada JM . How to reduce the incidence of placenta Accreta Spectrum independently of the number of cesarean? Maternal‐Fetal Medicine. 2019;1:68‐69.

[11] Roberge S , Demers S , Girard M , et al. Impact of uterine closure on residual myometrial thickness after cesarean: a randomized controlled trial. Am J Obstet Gynecol. 2016;214:507.e1‐507.e6. 10.1016/j.ajog.2015.10.916 26522861

[12] Antoine C , Pimentel RN , Reece EA , Oh C . Endometrium‐free uterine closure technique and abnormal placental implantation in subsequent pregnancies. J Matern Fetal Neonatal Med. 2021;34:2513‐2521. 31581865 10.1080/14767058.2019.1670158

[13] Vikhareva O , Rickle GS , Lavesson T , Nedopekina E , Brandell K , Salvesen KÅ . Hysterotomy level at cesarean section and occurrence of large scar defects: a randomized single‐blind trial. Ultrasound Obstet Gynecol. 2019;53:438‐442. 30484920 10.1002/uog.20184

[14] Soergel P , Jensen T , Makowski L , von Kaisenberg C , Hillemanns P . Characterisation of the learning curve of caesarean section. Arch Gynecol Obstet. 2012;286:29‐33. 22278149 10.1007/s00404-012-2230-9

[15] Silver RM , Fox KA , Barton JR , et al. Center of excellence for placenta accreta. Am J Obstet Gynecol. 2015;212:561‐568. 25460838 10.1016/j.ajog.2014.11.018

[16] Palacios‐Jaraquemada JM , Basanta NA . Does a more exact definition mean being able to reduce maternal morbidity in placenta accreta spectrum? Am J Obstet Gynecol. 2022;227:931‐932. 35841939 10.1016/j.ajog.2022.06.062

[17] Lyell DJ . Adhesions and perioperative complications of repeat cesarean delivery. Am J Obstet Gynecol. 2011;205:S11‐S18. 22114993 10.1016/j.ajog.2011.09.029

[18] Nieto‐Calvache AJ , Basanta N , Hussein AM , Rivera‐Torres LF , Palacios‐Jaraquemada JM . Paramedian access to the pelvis as a strategy in patients with bladder adhesions to the abdominal wall. Int J Gynecol Obstet. 2024;1‐3. Aug 10.1002/ijgo.15841 39087453

[19] Yabuki Y , Asamoto A , Hoshiba T , Nishimoto H , Nishikawa Y , Nakajima T . Radical hysterectomy: an anatomic evaluation of parametrial dissection. Gynecol Oncol. 2000;77:155‐163. 10739705 10.1006/gyno.1999.5723

[20] Palacios‐Jaraquemada JM , Basanta N , Nieto‐Calvache Á , Aryananda RA . Comprehensive surgical staging for placenta accreta spectrum. J Matern Fetal Neonatal Med. 2022;35:10660‐10666. 36543387 10.1080/14767058.2022.2154572

[21] Nieto‐Calvache ÁJ , Aryananda RA , Palacios‐Jaraquemada JM , et al. One‐step conservative surgery vs hysterectomy for placenta accreta spectrum: a feasibility randomized controlled trial. Am J Obstet Gynecol MFM. 2024;6:101333. 38458362 10.1016/j.ajogmf.2024.101333

[22] Bouchghoul H , Madar H , Resch B , et al. Uterine‐sparing surgical procedures to control postpartum hemorrhage. Am J Obstet Gynecol. 2024;230:S1066‐S1075. 37729440 10.1016/j.ajog.2022.06.018

[23] Muñoz M , Stensballe J , Ducloy‐Bouthors AS , et al. Patient blood management in obstetrics: prevention and treatment of postpartum haemorrhage. A NATA consensus statement. Blood Transfus. 2019;17:112‐136. 30865585 10.2450/2019.0245-18 PMC6476742

[24] Spahn DR , Muñoz M , Klein AA , Levy JH , Zacharowski K . Patient blood management: effectiveness and future potential. Anesthesiology. 2020;133:212‐222. 32108683 10.1097/ALN.0000000000003198

[25] Muñoz M . Patient blood management in obstetrics: a plea for widespread and effective implementation. Blood Transfus. 2024;22:4‐6. 38276913 10.2450/BloodTransfus.653 PMC10812891

[26] World Health Organization . The urgent need to implement patient blood management: policy brief. World Health Organization; 2021.

[27] Qureshi YA , Tai NRM . Surgical haemostasis. In: Thomas WEG , Reed MWR , Wyatt MG , eds. Oxford Textbook of Fundamentals of Surgery. Oxford University Press; 2016:543‐547.

[28] Cicinelli E , Einer‐Jensen N , Galantino P , Alfonso R , Nicoletti R . The vascular cast of the human uterus: from anatomy to physiology. Ann N Y Acad Sci. 2004;1034:19‐26. 15731296 10.1196/annals.1335.002

[29] Palacios Jaraquemada JM , García Monaco R , Barbosa NE , Ferle L , Iriarte H , Conesa HA . Lower uterine blood supply: extrauterine anastomotic system and its application in surgical devascularization techniques. Acta Obstet Gynecol Scand. 2007;86:228‐234. 17364288 10.1080/00016340601089875

[30] Sinav A . History of anatomical errors in anatomy textbooks and atlases. Int J Ment Health Psychiatry. 2018;4:34.

[31] Palacios‐Jaraquemada JM , Karochi M , Keith L . In: Arulkumaran S , Karoshi M , Keith LG , Lalonde AB , B‐Lynch C , eds. Uterovaginal Blood Supply: the S1 and S2 Segmental Concepts and their Clinical Relevance. A Comprehensive Textbook of Postpartum Hemorrhage. 2nd ed. Sapiens Publishing; 2012:19‐23.

[32] Palacios‐Jaraquemada JM , Nieto‐Calvache Á , Basanta NA . Anatomical basis for the uterine vascular control: implications in training, knowledge, and outcomes. Am J Obstet Gynecol MFM. 2023;5:100953. 37031866 10.1016/j.ajogmf.2023.100953

[33] Nieto‐Calvache AJ , Palacios‐Jaraquemada JM , Sarria‐Ortiz D , Galindo‐Velasco V , Basanta N . How to choose and apply a uterine compression suture for the management of postpartum hemorrhage? Int J Gynecol Obstet. 2024;166:902‐904. 10.1002/ijgo.15468 38469891

[34] Rathod S , Samal SK , Swain S . A three year clinicopathological study of cases of rupture uterus. J Clin Diagn Res. 2015;9:QC04‐QC06. 10.7860/JCDR/2015/14554.6783 PMC4668475 26673858

[35] Boyle JG , Gabbe SG . T and J vertical extensions in low transverse cesarean births. Obstet Gynecol. 1996;87:238‐243. 8559531 10.1016/0029-7844(95)00388-6

[36] Nieto‐Calvache AJ , Palacios‐Jaraquemada JM , Basanta N , et al. Internal manual compression of the aorta‐an effective way to temporarily control pelvic bleeding in obstetrical hemorrhage. Am J Obstet Gynecol. 2022;227:96‐97. 35248574 10.1016/j.ajog.2022.02.040

[37] Nieto‐Calvache AJ , Palacios Jaraquemada JM , Aryananda RA , et al. External aortic compression: buying time to save lives in obstetric hemorrhage. Am J Obstet Gynecol. 2024. doi:10.1016/j.ajog.2024.09.017 [Online ahead of print]. 39304012

[38] Habte A , Bizuayehu HM , Lemma L , Sisay Y . Road to maternal death: the pooled estimate of maternal near‐miss, its primary causes and determinants in Africa: a systematic review and meta‐analysis. BMC Pregnancy Childbirth. 2024;24:144. 38368373 10.1186/s12884-024-06325-1 PMC10874058

[39] Howell AM , Burns EM , Bouras G , Donaldson LJ , Athanasiou T , Darzi A . Can patient safety incident reports Be used to compare hospital safety? Results from a quantitative analysis of the English National Reporting and learning system data. PLoS One. 2015;10:e0144107. 26650823 10.1371/journal.pone.0144107 PMC4674095

